# Ice nucleation ability of loess from the northwestern United States

**DOI:** 10.1371/journal.pone.0220991

**Published:** 2019-08-09

**Authors:** Gourihar Kulkarni

**Affiliations:** Atmospheric Sciences and Global Change Division, Pacific Northwest National Laboratory, Richland, Washington, United States of America; Hong Kong University of Science and Technology, HONG KONG

## Abstract

The heterogeneous nucleation of ice processes involving loess particles that influences the formation of mixed-phase clouds are poorly understood. Here, the ice nucleating ability of wind-blown dust or loess accumulated from the past glaciated area was investigated at three temperatures: -26, -30, and -34 °C and at below and above saturation with respect to liquid water conditions. Total six loess samples from different regions across Columbia Basin province, WA, USA were collected, dry dispersed, size-selected at mobility diameter 200 nm, and investigated for their ice nucleation efficiency. To understand the effect of atmospheric processing during long-range transport on their ice nucleating ability, similar experiments were also performed on acid-treated loess samples. Additionally, the ice nucleating properties of Arizona Test Dust (ATD) were investigated as a surrogate for natural mineral dust particles to test the experimental approach. Results show that treated particles have lower ice nucleation efficiency compared to untreated particles at all temperature and saturation with respect to liquid water conditions. Comparison based on ice-active site density (*N*_*s*_) metric indicate that loess particles at saturation with respect to liquid water conditions are marginally more efficient than the mineral and soil dust values reported in the literature, but they have lower efficiencies than the predicted *N*_*s*_ efficiency of K-feldspar particles at supercooled temperatures greater than -38 °C.

## Introduction

Silt-sized terrestrial sediment or loess formed by the accumulation of wind-blown dust covers approximately 10% of the world’s land surface [1, 2]. Loess deposits are mostly concentrated at high-latitude, and the analysis of such fine particles allows us to better understand their role in the earth-atmosphere system [3–5]. This wind-deposited silt accumulated at ice flooded plains can be a significant source of atmospheric mineral dust amount. The burden of such mineral type in the atmosphere may affect aerosol-radiation, by interacting with solar radiation through absorption and scattering, and aerosol-cloud, by cloud properties and serving as ice nucleating substances, interactions. The loess mostly consists of feldspar, mica, quartz, illite, and calcite minerals, and the contribution of each constituent varies with the location of deposits [5]. The very fine grain (< 2 μm) fraction can be lifted to high altitudes and subjected to long-range transport [6, 7]. Atmospheric processing can further modify the physical and chemical properties of these emitted particles and affect the aerosol-cloud interaction [8, 9].

Previous studies concluded that individual, as well as a complex mixture of different, minerals are efficient ice nucleating particles (INPs) in different modes of ice nucleation [10, 11]. Heterogeneous ice nucleation can proceed through i) deposition mode: ice formation by direct water vapor deposition on INP; ii) immersion/condensation mode: freezing of a supercooled water or solution droplet, and iii) contact nucleation: freezing initiated when INP contacts a supercooled water droplet. Previously, many studies also investigated atmospheric aging effect by coating the atmospherically relevant mineral dusts (for eg. kaolinite, illite, K-feldspar) with soluble materials such as sulfuric acid and concluded that the INP efficiency of acid treated mineral dust particles is typically reduced compared to untreated particles largely because the coating modifies the surface properties [12–14]. However, ice nucleation studies that investigate the effect of acid coating that simulates the atmospheric aging for loess particles are missing.

In this paper, we report the first results from an experiment designed to investigate the ice nucleation efficiency of bare and acid coated loess from Columbia Plateau province of the northwestern United States. To understand the implications of this loess variability towards ice nucleation, bulk surface samples were collected from six sites distributed across the plateau. These samples were size-selected based on their mobility diameter, and investigated for their ice nucleation efficiency at various temperatures and humidity conditions. To understand the effect of atmospheric aging, particularly chemical aging where condensation of acids may occur while particles are transported in the atmosphere, the samples were treated with acids, and the ice nucleation efficiency of these acid treated particles was investigated.

## Materials and methods

### 2.1 Site location and sample collection

Six undisturbed surface samples of loess from Columbia Plateau province were collected. No specific permissions were required for these locations because they are public. This field study also did not involve endangered or protected species. The location of the sampling site was based on the map that shows loess distribution across the province [15, 16]. See S1 Fig and S1 Table for details regarding geographical locations of sampling sites. Furthermore, sampling from flat topography regions of loess was avoided as they provide fertile soil for agriculture, and therefore samples were collected from the terraces of small hills that are undisturbed by the agricultural activity. At each site, surface soils (0–10 cm depth) up to 50 grams within 1.0 m radial area were collected in sterile Whirl-Pak store bags, and the samples were stored in the freezer.

### 2.2 Particle generation and treatment

**Fig 1:**
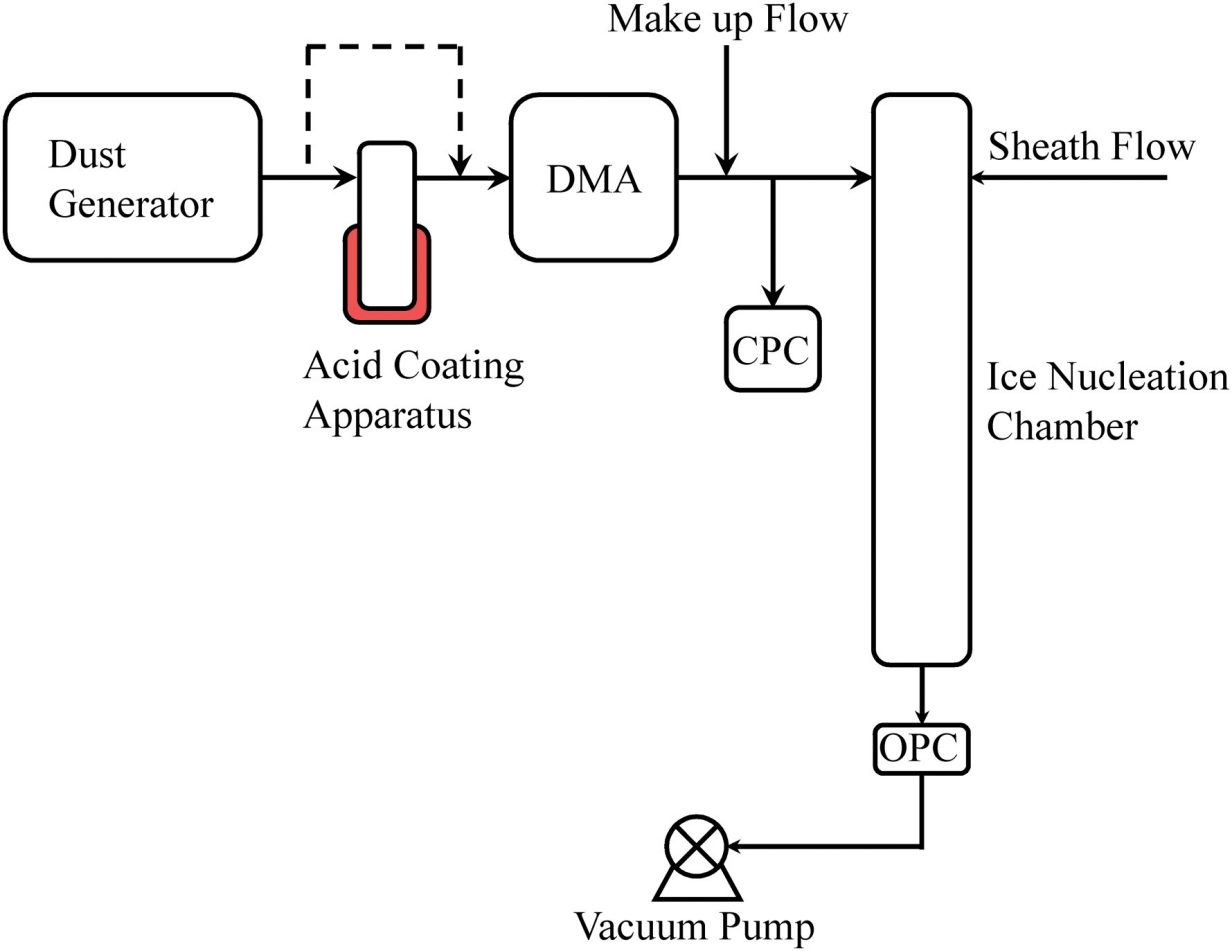
Experimental schematic to investigate the INP efficiency of loess particles. The particles are dry dispersed, size-selected based on their mobility diameter, and investigated for their ice nucleation efficiency. Three different particle treatment: untreated, treated with acid, and heat treated experiments were carried out, and the INP efficiency was calculated to understand the importance of treatment towards ice nucleation. Particles with a mobility diameter of 200 nm were selected in a DMA. CPC concentration was kept nearly similar in ATD and loess particle experiments. DMA: differential mobility analyzer; CPC: condensation particle counter.

Loess samples were sieved (Newark Superla) to eliminate any vegetation debris and particles larger than ~50 μm. Fig 1 shows a schematic diagram of the ice nucleation experimental set up. Each individual loess sample was dry-dispersed using a dust generator (TSI Inc., Model 3433), and these dispersed particles were size-selected based on their mobility diameter of 200 nm using a differential mobility analyzer (DMA; TSI Inc., Model 3080). A mobility of diameter of 200 nm was chosen because particles of this size were sufficiently high, and this higher number concentration of particles helped to improve the INP detection statistics. This particle selection methodology however may induce some uncertainty in determining the true nature of INP efficiency of loess particles because in the atmosphere entire size-distribution of loess particles may be available [17]. Further, these nearly monodisperse particles are transmitted to a condensation particle counter (CPC; TSI Inc, Model 3010) and ice nucleation chamber [14, 18]. Based on the theoretical calculations [19], the double (~325 nm) and triple (~445 nm) charged particles may be present within the aerosol stream, but the contribution of multiply charged particles is less than 10%. In acid coating experiments, the dry dispersed particles were passed through a coating apparatus maintained at 150 °C. The sulfuric acid with ACS reagent grade of 95–98% was used. More apparatus details are previously published [14], but briefly, the apparatus consists of a glass tube and a vacuum trap assembly that consists of the acid bath. The acid bath is heated using heating tapes, and the temperature of the bath is precisely controlled using an all-purpose digital temperature controller. A constant aerosol stream flow of 0.3 LPM was maintained that limited the residence time of the particles to ~ 5 s and coating thickness to ~ 40 nm [14]. To validate the operation of coating apparatus, the above experimental procedure was repeated but using size-selected ATD (Powder Technology, Inc) particles. Ice nucleation efficiencies of bare and coated ATD particles was compared. Further, the influence of only heating was investigated by operating the coating apparatus without any acid. Again the blank coating apparatus was operated at 150 °C, and these experiments are defined as heat treated. Heat treated experiments were carried out primarily at -34 °C because particles are more efficient in inducing nucleation of ice at colder temperatures. However, two experiments at each -26 and -30 °C temperatures were also performed to examine the consistency.

### 2.3 Ice nucleation measurements

For the ice nucleation experiments, the PNNL ice nucleation chamber was used. The chamber design and operating procedure have been discussed in several previous studies [18, 20–22]. Briefly, the ice chamber consists of two vertical parallel aluminum plates separated by a distance of ~10 mm that are cooled independently using a temperature controlled cooling bath (Lauda Brinkmann Inc.; New York, NY, USA). Both the walls of the chamber are coated with the ice layer (~0.3 mm), and to produce known ice-supersaturation conditions a linear temperature gradient is applied between the plates. These temperature measurements are further used to calculate the relative humidity with respect to ice (RH_ice_) and relative humidity with respect to water (RH_w_) using Murphy and Koop (2005) [23] vapor pressure formulations. The sample and sheath flow constitute the total flow rate within the chamber, which is 10 LPM, and therefore the residence time of the particles within the chamber is ~12 s. The sample flow rate is 1 LPM. The bottom of the chamber is attached with the evaporation section to evaporate the supercooled droplets and the section is maintained at aerosol lamina temperature conditions. The water droplet breakthrough RH_w_ limit is ~112%. Above this limit, the phase discrimination between ice crystals and supercooled droplets is not possible, and therefore all the experiments are terminated well below this limit when chamber reached RH_w_ = 110%. The RH_w_ and temperature uncertainties are calculated based on the temperature difference across the width of the aerosol lamina profile between the two plates. These uncertainties were determined using temperature values of cold and warm plates maintained at -40 and -20 °C, respectively, and chamber wall temperature uncertainties (~ ±0.2 °C). The calculations show that the uncertainty in the aerosol lamina temperature and RH_w_ are ~0.5 °C and ~2.5%, respectively. The particles that induced nucleation of ice and further grown to a size larger than ~2 μm are classified as ice crystals, which were measured by the optical particle counter (OPC; CLiMET, model: CI-3100). The ice active fraction was calculated as the ratio of number of ice crystals to the total number size-selected particles that enter the ice chamber. A blank experiment was performed using only filtered air to understand the background ice fraction, and it was carried out at the beginning and end of the experiment. The blank experiment ice fraction was ~ 0.01%, and this fraction was subtracted from the active fraction measured at each temperature. The ice nucleating abilities of loess particles was further calculated using ice-active surface site density (*N*_*s*_) [21, 24]. This approach allows to formulate a simple ice nucleation parameterization that can be used to compare against other measurements from the literature [8, 25]. It should be noted that *N*_*s*_ approach ignores the time dependency during the nucleation events, but describes the number of ice nucleation active sites distributed over the particle surface [26, 27]. The *N*_*s*_ can be calculated as

$$N_{s}=-\frac{1}{A}\;ln(1-AF)$$ (1)

where *AF* is the ice active fraction at RH_w_ = 109% and *A* is the surface area per particle. For the surface area calculations it is assumed that the particles are spherical in shape. Surface area calculations could be influenced by the presence multiple charged particles and particle shape [28, 29]. Assuming the monodisperse population and spherical shape of particles towards calculating the surface area may influence the *N*_*s*_ calculations. Such that the reported *N*_*s*_ values represent upper estimates of *N*_*s*_ values of loess particles. Further, the measurement uncertainties from *A* and *AF* quantities were combined using error propagation method to calculate the uncertainty in *N*_*s*_.

## Results and discussion

**Fig 2:**
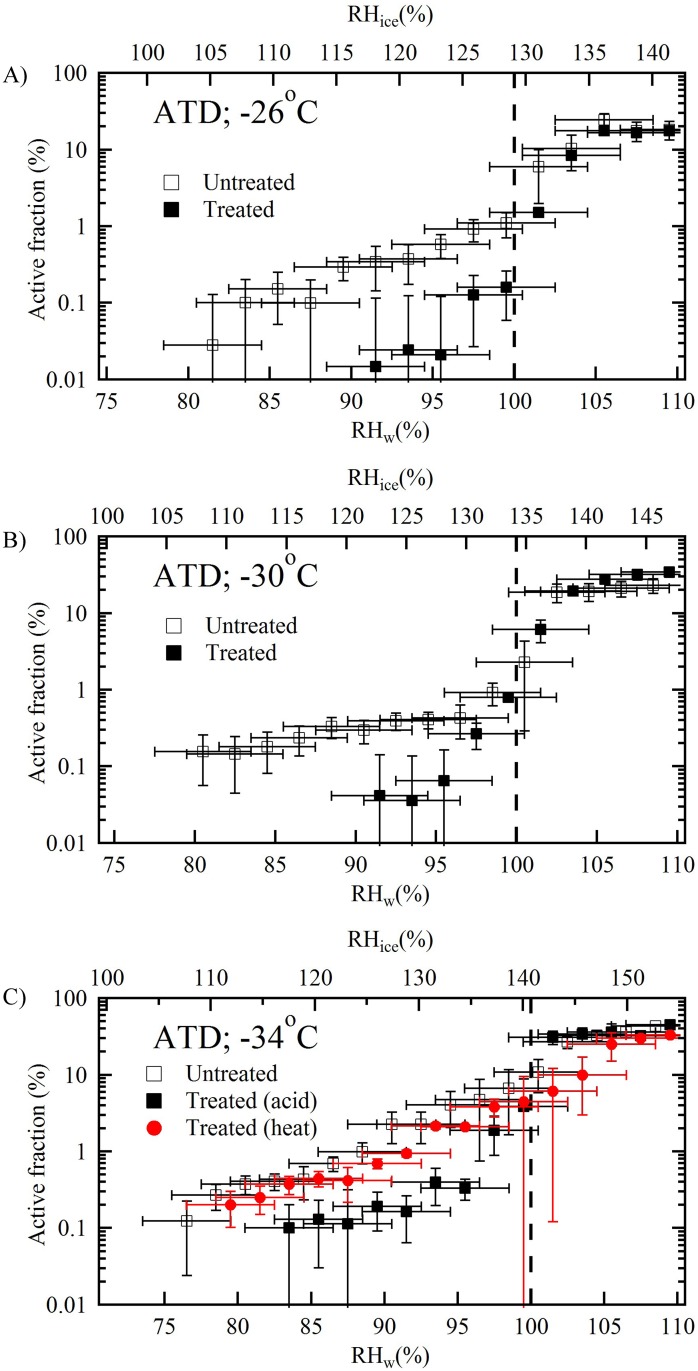
Ice active fraction of 200 nm untreated, treated, and heat treated ATD particles as a function of temperature and humidity conditions. The INP efficiency of heat treated particles is shown only at -34 (panel C). Vertical dashed line indicates the water saturation line. The vertical errors bars are equal to the one standard deviation of the measurements, and the horizontal error bars are equal to the ±2.5% uncertainty in the RH_w_ measurements.

Active fraction represents the measurement of ice nucleation efficiency of INPs. Fig 2 shows the activated fraction of ATD particles as a function of temperature and saturation with respect to liquid water conditions. Henceforth, saturation with respect to liquid water conditions will be referred to as saturation conditions. It was observed that ice nucleating ability of treated particles is significantly reduced at subsaturation conditions. Results from heat treated experiments (-34 °C), where no acid solution was used, show that the ice nucleating properties of untreated and heat treated particles are nearly similar.

**Fig 3:**
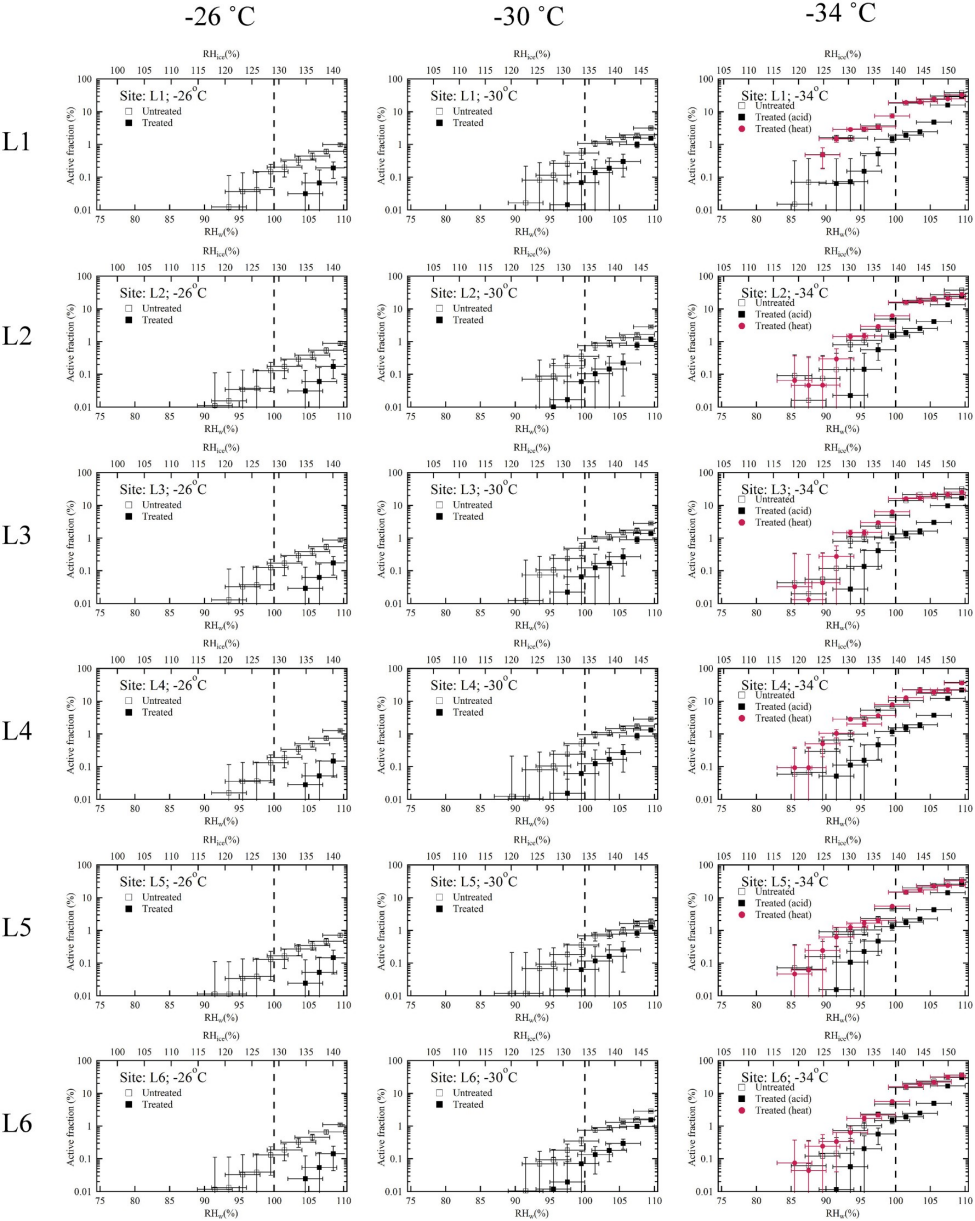
As in Fig 2, but for loess particles as a function of temperature and humidity conditions. Natural loess samples from six different locations (L1 to L6) from the Columbia Plateau province, WA, USA, were investigated to understand the ice nucleation efficiency at three different temperatures (-26, -30 and -34 °C).

Fig 3 shows the activated fraction of loess from different sites as a function of temperatures at various RH_w_ or RH_ice_. Ice nucleation of loess was observed in the deposition mode or subsaturated conditions; RH_w_ < 100% and immersion/condensation freezing mode or saturated conditions; RH_w_ ≥ 100%. It should be noted that deposition mode may be viewed as immersion freezing mode because surface voids and cracks of the loess could activate liquid water at RH_w_ < 100% and this water can freeze via immersion/condensation freezing mode [30]. For simplicity, the results are discussed in the context of saturation conditions and the use of specific ice nucleation mode is avoided. The vertical and horizontal error bars are equal to the one standard deviation of the measurements (n = 3) and uncertainty (± 2.5%) in the RH_w_ calculations, respectively.

**Fig 4:**
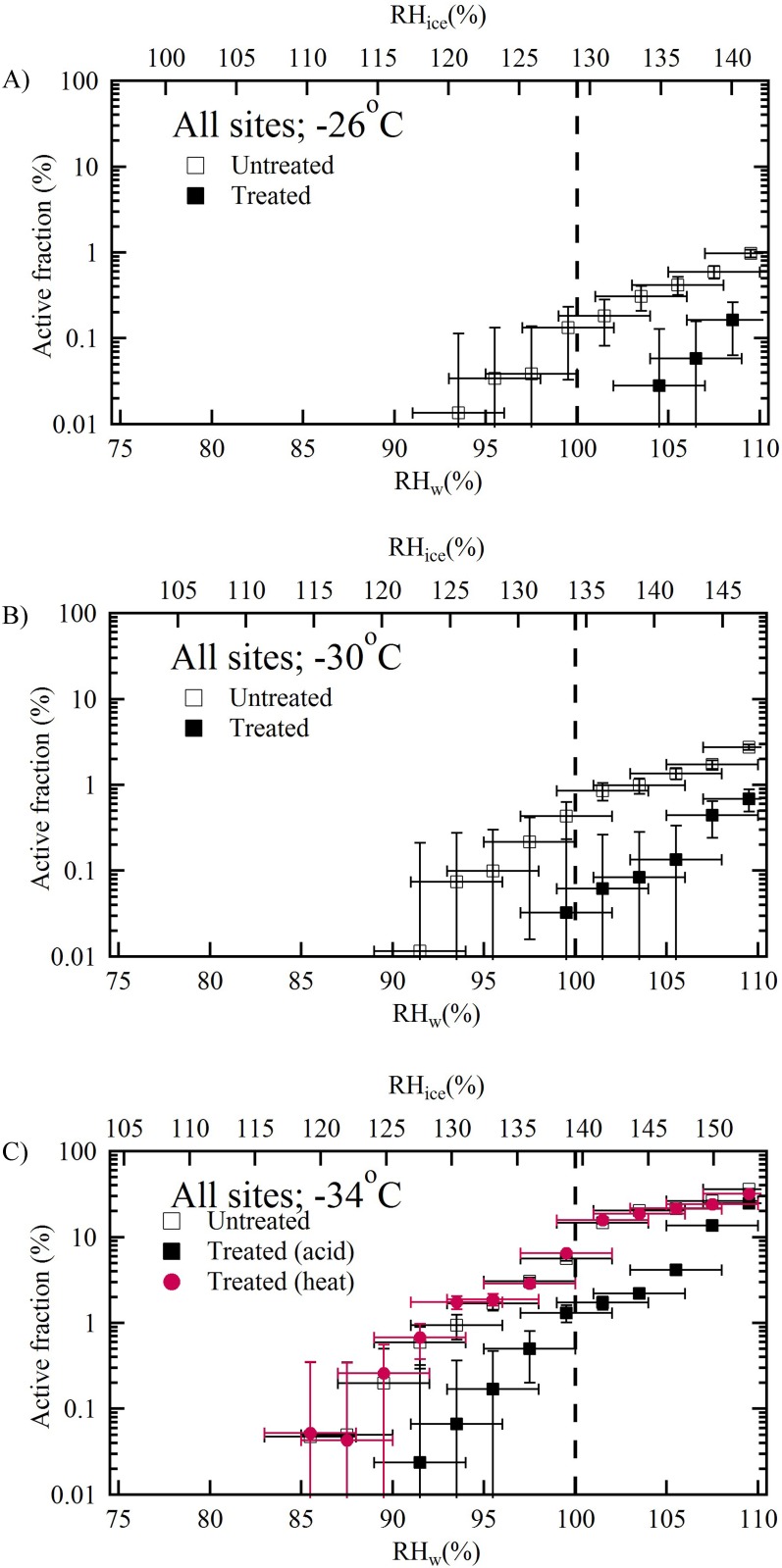
As in Fig 2, but shows average ice nucleating properties of loess particles from all six sites as a function of temperature and humidity conditions. The ice nucleation efficiency of loess measured at six sites is averaged as a function of temperature (-26, -30 and -34 °C).

To best represent the ice nucleating behavior of loess, the active fraction data from each site was combined, and the average active fraction was calculated as a function of temperature as shown in Fig 4. Each panel (Fig 4A to 4C) shows the average ice nucleating properties from six sites at different temperatures (-26, -30 and -34 °C). The results show that the active fraction increases as RH_w_ increases. At subsaturated conditions treated particles have lower ice nucleation efficiency compared to the untreated particles at various temperatures. The onset of RH_w_ conditions: the minimum RH_w_ required to observe ice nucleation on untreated and treated particles, varied with the temperature. At -26 °C, the ice nucleation on the untreated and treated particles occurred at 94% and 104% RH_w_ conditions, respectively, suggesting saturated conditions are required for the treated particles. The onset RH_w_ was slightly reduced at -30 °C. Ice nucleation was observed at 92% and 100% RH_w_ conditions. The onset was further reduced at a lower temperature (-34 °C) where ice nucleation was observed at 86% and 92% RH_w_ conditions. The maximum active fraction reached 109% RH_w_ conditions and, at each temperature: -26, -30 and -34 °C for untreated particles it was 1, 3 and 36%, respectively, and for treated particles it was 0.2, 0.7 and 24%, respectively. These results indicate the ice nucleation efficiency increases with decrease in temperature and the ice nucleating ability of loess reduces after coating. The effect of heating the particles within the acid coating apparatus might inadvertently affect the ice nucleation efficiency. However, results show that only heating the particles does not affect the original (untreated) ice nucleation efficiency of particles. It was observed that the active fraction of untreated and untreated but heated samples were nearly similar at all RH_w_ conditions. Similar conclusions were also derived by Sullivan et al. (2010) [31]. They reported similar ice nucleating abilities of unheated and heated ATD particles. In their experiment thermodenuder maintained at a temperature 250 °C was used to heat the particles. The residence time of particles in their set up was 4 to 5 s; residence time within the current study is ~5 s. Effect of only heat treatment on ice nucleation efficiency was not discernable because it is likely that surface physical and chemical sites might have remained unaltered [12] and organic matter was not completely lost [32]. However, it should be noted that biological INPs are sensitive to the heat treatments and their reduced contribution after treatment could affect the ice nucleation efficiency [33]. These current loess samples seems to lack the biological particles and therefore the ice nucleation efficiencies were not affected by the heat treatment.

The suppression in the ice nucleating ability of treated loess was observed and dependent on the temperature and saturation conditions. The reduction in ice nucleating ability at -26 °C was so severe that ice nucleation was only observed at saturation conditions. Previous ice nucleation experiments with acid processing of mineral dust particles have concluded similar observations (e.g. Tang et al. 2016 and references therein) [8]. Deactivation of ice nucleating ability at below saturation conditions was reported for acid treated ATD [14, 31, 34], kaolinite and montmorillonite particles [35–37]. Above saturation conditions the treated loess also exhibited lower ice nucleating ability compared to the untreated particles. Sullivan et al. (2010) [31] and Kulkarni et al. (2014) [14] report that ice nucleating abilities of acid treated ATD particles are not suppressed in saturation conditions. However, Niedermier et al. (2011) [13] and Tobo et al. (2012) [36] reported the complete suppression at various temperature conditions warmer than -30 °C at saturation conditions, but at colder temperatures, no significant differences were observed between untreated and treated particles.

The reduction in the ice nucleation efficiency could be attributed to the modification of the untreated particle surface properties, the strength of an acid, exposure time of the particles to acid, and accumulation of acid reaction products on the particle surface. Sullivan et al. (2010) [31] surmise that the formation of acid reaction products impaired the ice nucleating abilities at below saturation conditions, but a dissolution of these products at saturation conditions exposed ice nucleating sites, and therefore no reduction in ice nucleating ability was observed at saturation conditions. Niedermeier et al. (2011) [13] and Reitz et al. (2011) [38] report that the reaction products such as aluminosilicates and ammonium sulfates can form and affect the ice nucleation behavior. Wex et al. (2014) [39] suggest that feldspar surfaces, which are efficient at inducing nucleation of ice, after reacting with the acid may transform into a poor ice-nucleating material. Kulkarni et al. (2014) [14] performed spectroscopic studies of acid treated particles and concluded that surface structural order (lattice constants) of these particles are modified compared to the untreated particles. Sihvonen et al. (2014) [35] observed reaction products such as hydrated aluminum sulfate and structural changes at the mineral surfaces. They concluded that these morphological and chemical changes might be responsible for the reduction in the ice nucleation efficiency. Structural changes could be sensitive to the strength of acid such that stronger acids can leach/react with the interlayer structure of dust particles [40, 41]. Past acid treatment ice nucleation studies also report that the impact of the coating depends upon the composition of mineral dust particles and coating thickness. Wex et al. (2014) [39] concluded that acid coated kaolinite particles had reduced ice nucleation efficiency compared to untreated particles probably because of alteration of surface properties responsible for inducing nucleation of ice. Further, Kulkarni et al. (2014) [14] reported a reduction in ice nucleating ability of coated illite, montmorillonite, and K-feldspar particles at only subsaturation conditions and no influence of acid coating thickness (up to 40 nm). The discussion of these studies suggests that several reasons could influence the ice nucleation properties of acid treated particles and motivate further investigations to understand the details of these reactions.

**Fig 5:**
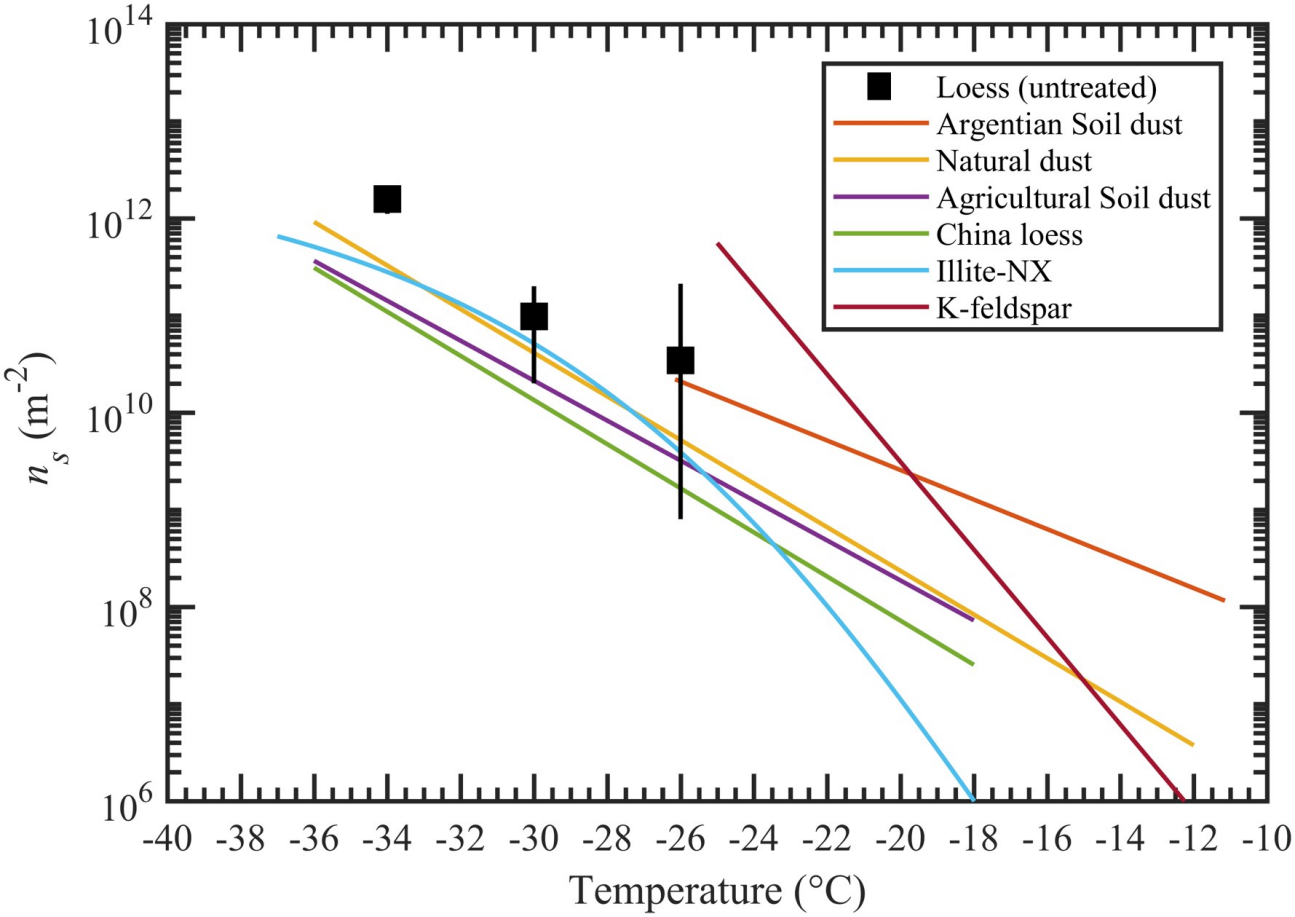
Surface active-site density parameter, *N*_*s*_, data calculated using 200 nm spherical particle diameter and ice active fraction at ~109% RH_w_ reported in Fig 4. See text for details. *N*_*s*_ was compared against various mineral dust and soil parameterizations taken from the literature. The parameterizations for Argentian soil dust, natural dust, agricultural soil dust, China loess, illite-NX, and K-feldspar were taken from Steinke et al. 2015 [42], Niemand et al. 2012 [25], Tobo et al. 2014 [32], Tobo et al. 2014 [32], Hiranuma et al. 2015 [43], and Atkinson et al. 2013 [44], respectively.

The ice nucleation efficiency with which loess nucleates ice are also described by the *N*_*s*_. The *N*_*s*_ parameter has been widely used to compare ice nucleation results between different measurement techniques and ice nucleating substances. Fig 5 shows the *N*_*s*_ values calculated using average active fractions (Fig 4) observed at the saturation conditions (RH_w_ = 109%), and they varied from ~7×10^10^ to 2×10^12^ m^-2^ with the temperature between -26 to -34 °C.

The *N*_*s*_ values for loess are consistent with previous studies, who studied different deserts dust and natural soils except at colder temperature where loess values are at least one order magnitude higher. Direct comparison with a parameterization of K-feldspar is not possible because it needs to be extrapolated to the lower temperature (< -25 °C). However, recently Niedermeier et al. (2015) [45] showed leveling off of *N*_*s*_ curves for K-feldspar at temperatures warmer than -38 °C dependent upon the particle surface area and K-feldspar content in the desert dust. Assuming 25% of K-feldspar they calculate *N*_*s*_ ~ 2×10^12^ m^-2^ as an upper limit at a temperature below ~-32 °C. This maximum derived value is comparable to the *N*_*s*_ calculated for loess sample at -34 °C suggesting a similar fraction of K-feldspar content may be possible in loess samples. Future experiments to characterize the physio-chemical properties of loess samples are required to better understand the atmospheric implications and representation in cloud models.

## Summary and conclusions

The paper reports the ice nucleation efficiency of six natural loess samples from the Columbia Plateau province, WA, USA. The samples were dry dispersed, and the ice nucleation efficiency of size-selected particles at various temperatures (-26, -30 and -34 °C) above and below saturation conditions was measured. To understand the implications of atmospheric aging on ice nucleation efficiency, the similar experiments were also performed but using acid treated particles. It was observed that the ice nucleating ability of treated particles was reduced at all temperature and saturation conditions. At -26 °C, treated particles required saturation conditions to induce nucleation of ice. At other temperature conditions, the treated particles were efficient at subsaturation conditions, but they required high RH_w_ (> 90%) to nucleate ice. Comparison of ice nucleating ability with the literature data for natural dust and soils shows that the untreated loess samples are slightly more efficient. The *N*_*s*_ values of loess samples are at least one order of magnitude higher at -34 °C, but within the uncertainty limit at other two temperatures. Future experiments are needed to investigate the composition of untreated and treated samples to better understand the ice nucleating properties of loess.

## Supporting information

S1 Data Minimal data set to verify the experimental setup. (XLSX)

S1 Fig Google. (n.d.). [Google Map showing six sampling sites within Washington State, USA]. Retrieved July 3, 2019 from https://goo.gl/maps/DNXKNaxG2d25yBLYA.
(TIF)

S1 Table The geographic coordinates of six sampling locations. (TIF)
