# Supplementary figures and images for: Ice nucleation ability of loess from the northwestern United States
Source: PLoS One. 2019 Aug 9;14(8):e0220991. doi: 10.1371/journal.pone.0220991 (PMC6688799; doi:10.1371/journal.pone.0220991)

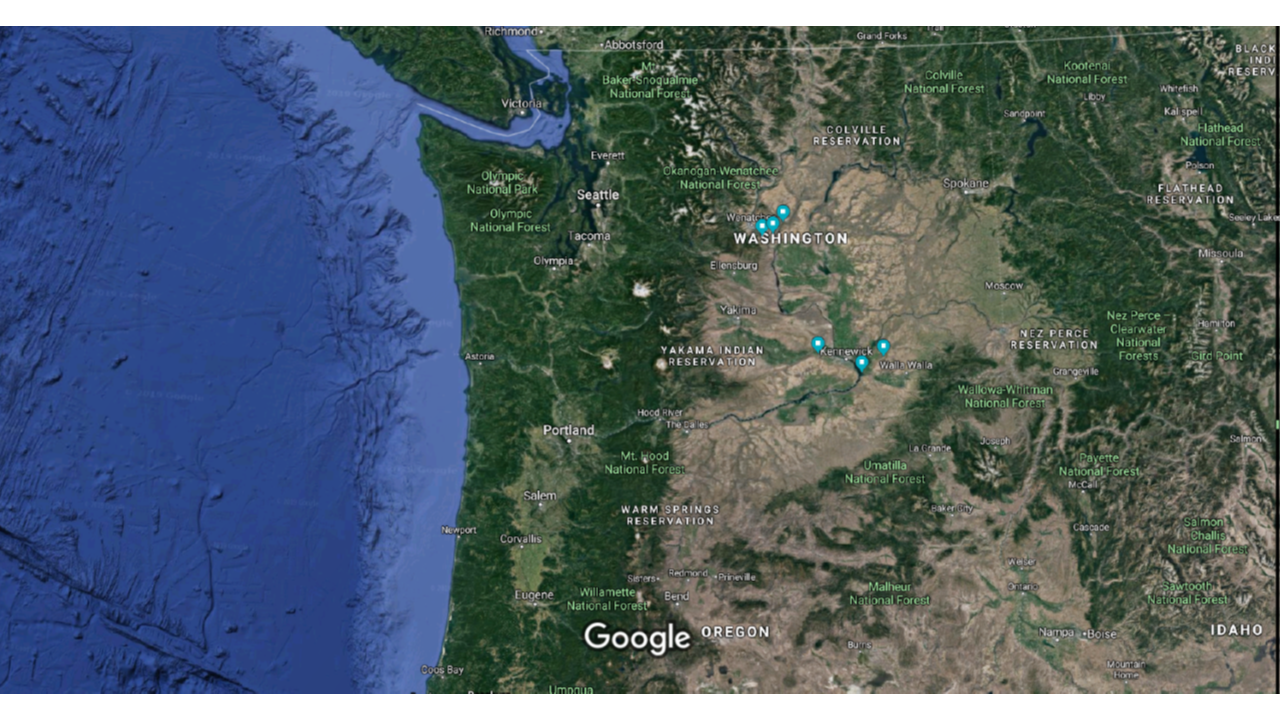

Supplement: S1 Fig — [Google Map showing six sampling sites within Washington State, USA]. Retrieved July 3, 2019 from https://goo.gl/maps/DNXKNaxG2d25yBLYA. (TIF) [file pone.0220991.s002.tif]

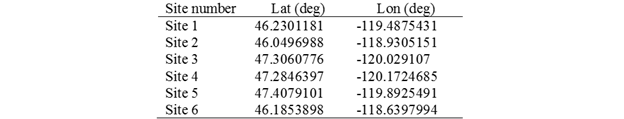

Supplement: S1 Table — (TIF) [file pone.0220991.s003.tif]
